# Against Strong Ethical Parity: Situated Cognition Theses and Transcranial Brain Stimulation

**DOI:** 10.3389/fnhum.2017.00171

**Published:** 2017-04-11

**Authors:** Jan-Hendrik Heinrichs

**Affiliations:** Forschungszentrum Jülich, Institute of Neuroscience and Medicine, Ethics in the Neurosciences (INM-8)Jülich, Germany

**Keywords:** situated cognition, ethical parity, extended mind, trancranial brain stimulation, neuroethics

## Abstract

According to a prominent suggestion in the ethics of transcranial neurostimulation the effects of such devices can be treated as ethically on par with established, pre-neurotechnological alterations of the mind. This parity allegedly is supported by situated cognition theories showing how external devices can be part of a cognitive system. This article will evaluate this suggestion. It will reject the claim, that situated cognition theories support ethical parity. It will however point out another reason, why external carriers or modifications of the mental might come to be considered ethically on par with internal carriers. Section “Why Could There Be Ethical Parity between Neural Tissue and External Tools?” presents the ethical parity theses between external and internal carriers of the mind as well as neurotechnological alterations and established alterations. Section “Extended, Embodied, Embedded: Situated Cognition as a Relational Thesis” will elaborate the different situated cognition approaches and their relevance for ethics. It will evaluate, whether transcranial stimulation technologies are plausible candidates for situated cognition theses. Section “On the Ethical Relevance of Situated Cognition Theses” will discuss criteria for evaluating whether a cognitive tool is deeply embedded with a cognitive system and apply these criteria to transcranial brain stimulation technologies. Finally it will discuss the role diverse versions of situated cognition theory can play in the ethics of altering mental states, especially the ethics of transcranial brain stimulation technologies.

## Introduction

The most widespread ethical concern regarding brain-stimulation technologies, be they invasive or transcranial, results from their potential of modifying the mind. Brain stimulation technologies have been likened to traditional psychosurgery, and just like the latter, their success and abuse already has a history of exaggeration^1^. The comparison creates an image that does carry.

In order to banish this image and highlight the pressing ethical issues in brain stimulation technology it has been suggested to treat the effects of such devices as ethically on par with established, pre-neurotechnological alterations of the mind, such as psychotherapy or mnemonics (Levy, 2007a,b; Fenton and Alpert, 2008). This parity is allegedly supported by situated cognition theories showing how external devices can be part of a cognitive system. I will portray these suggestions for revising the neuroethics of brain stimulation in section Why Could There Be Ethical Parity between Neural Tissue and External Tools?

It has, however, been doubted whether brain stimulation really can be seen as a part of a cognitive system, because it does not in fact fit the criteria of most situated cognition theories (Walter, 2009), much less the special case of *extended cognition*. In section Extended, Embodied, Embedded: Situated Cognition as a Relational Thesis I'll elaborate what in situated cognition approaches has been taken as a means for modifying the neuroethics of brain stimulation. I'll point out relevant limitations for such a use as well.

Drawing on situated cognition approaches, several sets of criteria for evaluating whether a cognitive tool is deeply embedded with a cognitive system have been suggested. In section On the Ethical Relevance of Situated Cognition Theses I will apply these criteria to transcranial brain stimulation technologies (tBS) and discuss the role diverse versions of situated cognition theory can play in the ethics of altering mental states, especially the ethics of tBS technologies.

## Why could there be ethical parity between neural tissue and external tools?

The debate about the role of situated cognition hypotheses in ethics started with the suggestion that the *extended mind* hypothesis might shed new light on neuroethical issues. I'll go into the details of situated cognition hypotheses in the next section, for now let it suffice to say that “situated cognition” is a collective term for several related paradigms in cognitive science: embodied cognition, embedded cognition, extended cognition or mind. They claim that the mind or cognition is dependent upon or constituted by more than brain tissue and its activity. In Levy (2007b) Levy claims that given the extended mind thesis the intuition becomes dubious “that neurological interventions, whether by way of psychopharmaceuticals, transcranial magnetic stimulation, or direct brain stimulation, are fundamentally different from more traditional ways of altering mental states” (Levy, 2007b, p. 7). It is surprising to see tBS, a technology which relies on the idea that cognition goes on in the brain and that by stimulating the brain we can improve cognition brought into close contact with approaches claiming that cognition happens not just in the head. Unsurprisingly, these examples, pharmaceuticals and brain stimulation technology, are not the focus of any situated cognition theorist. I will try to show that the neglect of brain stimulation examples in situated cognition theory has systematic reasons. While I share Levy's view that there is equal reason to be concerned about modifications of mental states independently of the specific vector, I wonder from which version, if any, of situated cognition this results.

Levy tries to support the case against internalist intuitions which posit an ethical difference between internal and external alterations of the mind by pointing not only to the extended mind hypothesis of Clark and Chalmers (1998), but to versions of the *embodied cognition* hypothesis as put forward by Damásio (1994), as well. He takes Damasio's neuroscientific results to show that the “mind extends into the body” (Levy, 2007b, p. 9). In his book length treatise (Levy, 2007a) he elaborates the claim of ethical parity between different modifications and carriers of mental states. He suggests an ethical parity thesis, providing a strong version, which relies on the theory of extended mind, and a weak version which makes use of the embedded mind theory only. These versions read:

EPP (strong): Since the mind extends into the external environment, alterations of external props used for thinking are *(ceteris paribus)* ethically on a par with alterations of the brain.
EPP (weak): Alterations of external props are *(ceteris paribus)* ethically on a par with alterations of the brain, to the precise extent to which our *reasons* for finding alterations of the brain problematic are transferable to alterations of the environment in which it is embedded (Levy, 2007a, p. 61).

As can be seen in the formulation of these two versions, the difference between different situated cognition approaches, i.e., extended mind and embedded cognition, finds explicit consideration. While Levy himself accepts the extended mind hypothesis and thus strong EPP, he is willing to settle for the weaker version in his discussion of neuroethical issues throughout the book^2^. Note however, that Levy's preferred version, EPP (strong) is enthymematic. It requires an additional premise, which might read “all contributors to the workings of a mind are on par with regard to their moral value, e.g., claim to protection” or “alterations to any contributor to the workings of the mind are ethically on par.” I prefer the former formulation as it highlights the moral value of external contributors to the mind, which situated cognition hypotheses are employed to support. The latter formulation is equivalent but highlights the moral standing of the alterations of said contributors. Without such a premise the argument would not be valid. I hope to show that there are serious reasons to doubt this hidden premise. EPP (weak) in contrast is not in need of an additional premise as it already contains the requirement that our moral reasons are transferable.

The parity principle is explicitly introduced with regard to psychopharmaceuticals and brain stimulation technologies and applies to all neurotechnologies in Levy's monograph^3^. In a later article Levy is considerably more careful in picking his examples. He explicitly states that the extended mind hypothesis is driven by new technologies, such as “brain-computer interfaces which expand their users' cognitive powers” but concedes that “it may be that the kinds of technology envisaged here prove to be beyond the capabilities of science for the foreseeable future” (Levy, 2011, p. 287 f.).

In parallel to Levy's contributions, Joel Anderson formulated a similar claim namely “that the supposition that the skin-and-skull barrier is a relevant ethical watershed […] involves bad metaphysics, and it has unacceptable ethical implications” (Anderson, 2008, p. 264)^4^. Anderson's primary focus is on prosthesis and tools used by persons with disabilities and in possible cases of enhancement. His use of enhancement examples transports an additional thesis he shares with Levy: neither the skin-and-skull barrier nor the therapy-enhancement distinction carry ethical weight. Rather the technologies in question need to be evaluated according to independent ethical criteria.

Anderson's version of a parity principle claims that two technological props are *ceteris paribus* ethically on par, if they are experientially equally transparent and functionally equivalent (Anderson, 2008, p. 266; but see the discussion of complementarity and functional eqivalence below). Whether they are located inside or outside the user's body does not play a significant ethical role. In arguing for this parity principle, Anderson takes recourse to situated cognition approaches, just as Levy does. It is, however, not clear which version of these approaches he relies on, as his position is compatible with the thesis of embodied cognition, embedded cognition as well as with the extended mind thesis in content and formulations. He refers to a person's cognitive processes as embodied (p. 262, 265), embedded (p. 264)^5^ or extended (p. 264 f.).

Although Anderson does not explicitly claim ethical parity between unmodified biological carriers of cognition and technological functional equivalents, this thesis seems to follow from his further arguments. He insists that we do not respect the embodiment of a person but the person herself. Accordingly, he would probably accept a stronger parity thesis, to the effect that any two carriers of cognitive processes are *ceteris paribus* ethically on par, if they are experientially equally transparent and functionally equivalent. According to this version of a parity thesis it does not matter whether the two carriers of cognitive processes are technological props or biological tissue.

Both Levy and Anderson deny that the distinction “inside the body/outside the body” *per se* has ethical relevance. Neither of them claims, however, that the risks associated with invasive procedures are ethically irrelevant. Both rightly insist on the opposite: if the same effect can be realized by a low risk procedure or a high risk procedure, which most invasive procedures are cases of, the prior is ethically preferable.

The consequences of these ethical parity theses for the ethics of neurotechnologies might be extensive. Would they for example imply that taking away Otto's famous notebook (Clark and Chalmers, 1998, see below, chapter 2.2) and destroying the part of the brain that still remembers to use the notebook are ethically on par? Do we have to infer that covering the papers you are doing sums on and virtual lesioning of your calculating brain by TMS are? Doubting that conclusion is at least possible. Below I will not just elaborate why situated cognition theses do not give rise to ethical parity but also why ethical parity is at best a rare exception. But first let me provide a short but telling example of how the ethical parity theses have been received and used in bioethical deliberation.

### The reception of the parity thesis in ethical deliberation

The ethical parity thesis has found extended application in its authors' own work, especially in Levy's introductory book *Neuroethics*. It has made an impact in a wide range of neuroethical articles, but finds only little explicit mention. For example, soon after Levy's work, Fenton and Alpert suggested that a specific case of neurotechnology should be considered as a part of the extended mind of its user and be ethically evaluated as such: Brain Computer Interfaces (BCI) for locked-in syndrome. While the example of neurotechnology is dissimilar to the technologies discussed in the present article, their reference to the parity principle and situated cognition approaches is typical for the debate.

Fenton and Alpert do refer to Levy's discussion of the extended mind hypothesis but not to the parity principle. Like Levy's, their reliance on situated cognition hypotheses is fairly inclusive. Unlike Levy they do not intend it as a forceful endorsement of some sort of situated cognition hypothesis. Rather they use situated cognition theories to modify a common perspective on technologies which closely interact with the (human) brain: “As with other embodied or embedded theories of cognition, extended mind theory can be regarded as a lens through which we learn to re-see particular aspects of human cognitive engagement with the relevant physical or social environment” (Fenton and Alpert, 2008, p. 126).

Their ethical conclusion on the moral value of BCIs follows established paths of bioethical enquiry. Rather than discussing whether BCIs should be treated as having the same moral value as biological means of cognition and motion, they focus on questions of informed consent in BCI surgery and distributional issues, namely society's duty to provide access to BCIs for persons in need of such. Their suggestion that such a societal duty exists, is presented as following from the improvements in autonomy and quality of life that persons with locked-in syndrome can gain from BCIs. Thus, the ethical parity thesis does not play any role in their argument.

Although Fenton and Alpert explicitly refer to situated cognition theses, these do not play an irreplaceable role in their discussion, either. The central reason for guaranteeing access to BCIs and for adapting a slightly modified informed consent procedure for BCIs Fenton and Alpert provide is the gain in autonomy of patients. This gain in autonomy, including the extended action space, or so they claim, changes the “self-nature” of the person. But one does not need to quote the extended mind hypothesis to establish that individuals with broader action space and more autonomy will lead different lives, think different thoughts and perform different actions. And neither does the evaluation that more autonomy and a broader action space are better for the person in question rely on the extended mind hypothesis. The only argumentative role it plays is to make these changes in the patient's characteristics a part of his “self-nature,” but that in turn does not carry any argumentative weight. Fenton and Alpert's results are sound without them making any argumentative use of either situated cognition theses or of the parity thesis.

In a direct reaction to Fenton and Alpert, Walter (2009) took care to differentiate the alternative versions of situated cognition hypotheses and demonstrated that BCIs for locked-in syndrome are not a case of extended cognition^6^. I suggest something similar is true for tBS. Walter rightly insists that ethical analysis, such as Fenton and Alpert's often do not require the use of situated cognition hypotheses. If, however, ethicists refer to such hypotheses, they should make their argumentative role very clear, and avoid the suspicion of mere window-dressing.

## Extended, embodied, embedded: situated cognition as a relational thesis

It is a family of innovative paradigms in cognitive science that the abovementioned authors base their ethical claims on. In order to evaluate whether these paradigms can support ethical claims beyond mere window-dressing, some in depth exposition of their structure is required. As mentioned, “situated cognition” is the collective term for different theses claiming that cognition and other mental processes can only be explained with regard to their bodily or environmental situatedness. The most common versions of situated cognition are embodied cognition, embedded cognition, extended mind, and enactivism. The various versions of situated cognition as new paradigms in cognitive science typically resulted from dissatisfaction with certain characteristics of the representational, computational paradigm in so called *standard* cognitive science (Shapiro, 2011). The reception as a metaphysical thesis in the philosophy of mind is secondary to that^7^. Its role in neuroethics, as it will turn out in the following, is even more modest.

Most versions of situated cognition theses have been put forward in the form of predications of “mind” or “cognition,” e.g., “extended mind,” “embedded cognition.” What these predications highlight is a *relation* between cognitive processes and complex organisms in an environment. One can thus differentiate the versions of situated cognition according to two dimensions: the type of relation and the relata. The relations between cognitive agent and their embeddings which are most commonly taken to hold, are dependency and constitution. The focus on organisms and their environment can vary strongly in scope. It can stop at the bodily limits of the organism, include his direct physical environment or even include his social and cultural embedding. Qua ethical parity thesis all of them would be candidates for moral value on par with that of our biological make-up. The varying scope of relata gives rise to one of the main desiderata of situated cognition hypotheses:

*D.: Situated cognition approaches need to determine the limits of how far cognition / the mind extends in space and time*.

Because situational factors are not merely candidates for being part of the human mind but qua parity thesis for equal moral status as our biological makeup, this desideratum is one of ethical theory as much as of cognitive science. In this chapter I will first provide an overview of the different versions of situated cognition theses (2.1), then I will disentangle the relations put forward in the different versions (2.2) and finally turn to the relata (2.3).

### A taxonomy of situated cognition theses

The overview of the main versions of situated cognition hypotheses will cover embodied, embedded and extended cognition theses. Enactivism does not play a role in the current debate about ethical parity.

The thesis of *embodied cognition* highlights that the cognitive processes of humans (and other animals) can only be explained as either dependent upon or partially constituted by extracranial bodily processes. Walter introduces Embodied Cognition I and II and distinguishes them by the relation between cognitive processes and extracranial bodily processes. To make the latter difference obvious:

“Embodied Cognition I (EC-I): Cognitive processes are partially dependent upon extracranial bodily processes” (Walter, 2009, p. 63). “Embodied Cognition II (EC-II): Cognitive processes are partially constituted by extracranial bodily processes” (Walter, 2009, p. 64).

The embodiment thesis has historically been a reaction to an alleged tendency to ignore the role of the body in explaining cognition, shown by the cognitive sciences under the sway of the computational metaphor^8^. It has for example been claimed, that visual perception is much closer integrated with bodily movement and bodily feedback, such as change of perspective and grasping behavior than the computational model was able to explain (Hurley, 1998; Noë, 2004).

The thesis of *embedded cognition* connects to that of embodied cognition type I and extends (no pun intended) one relatum of the dependency relation. It emphasizes that cognitive processes depend on extrabodily components or processes. Here is Walter's definition:

“Embedded Cognition (EMC): Cognitive processes are partially dependent upon extrabodily processes” (Walter, 2009, p. 64).

Embedded cognition approaches have been brought forth against the representational paradigm of standard cognitive science rather than against computationalism. They intended to show that representations are not necessary in explaining even prime examples of apparently representation-dependent tasks, such as spatial navigation^9^.

Finally, the thesis of the *extended mind* extends the relatum as before, from bodily to extrabodily processes and replaces the dependency relation by a constitution relation. Extended cognition approaches are well supported by, if not dependent on, embodiment approaches and vice versa. Thus, the separate treatment in the above and following passages is intended for analytic clarity, not to give the impression of mutually exclusive alternative positions. According to the thesis of,

“Extended Cognition (EXC): Cognitive processes are partially constituted by extrabodily processes” (Walter, 2009, p. 65).

While several variations of the extended cognition or extended mind hypothesis have been suggested (e.g., Rowlands, 1999), the most prominent version is that of Clark and Chalmers (1998). What makes their version one of the most ambitious is that they extend their claim to the mind in general, explicitly not limiting it to *cognitive* processes.

On the basis of this short overview of situated cognition theses, I will be able to differentiate between two types of moral value, primary and derivative, which technological systems for the support or modification of the mind, such as transcranial stimulation technologies might have. In order to do so I will need two additional conceptual clarifications. If the different situated cognition and situated mind approaches just presented are read as relational theses, we should be clear about two questions: (1) what exactly is it that is extended, embedded, embodied? This question targets the relata. And (2) what exactly is this relation of dependence or constitution? This question targets the relation. I'll provide my understanding of the latter question first and then turn to the more crucial, former question.

### The relations: constitution, dependency, and the risk of explanatory and ethical bloat

As mentioned the aim of investigating the structure of situated cognition theses here is to evaluate whether they justify treating transcranial stimulation technologies as ethically on par with our biological constituents of the mind. It will make a relevant difference in evaluating any technological support or modification of mental processes whether the former can be understood as *real parts* of the cognitive agent, as a necessary *scaffold* of an agent's cognitive processes or as mere *tools* of cognition. This difference in turn strongly depends on what one takes constitution and dependency to be, and how far the constitution or conditions of cognition extend.

I take “dependency” to refer to a causal, difference-making relation. Both embodied and embedded cognition theories want to claim that the body or the environment have an indispensable causal role in cognition. They cannot be content with any less ambitious claim according to which the body or environment are some non-causal condition for cognition. Causal dependency is for the present purpose captured sufficiently in the INUS-conditions following Mackie (1965). “Constitution,” on the other hand, will in the following be understood as a relation which obtains between wholes and the sum of their parts and the relations between those parts^10^. Even this minimal characterization will suffice to clarify the difference between causal dependence and constitution and elucidate how this difference plays out in the parity principle.

First, constitution is a relation between entities that are not wholly distinct, such as parts and a whole, or the material and its form as e.g., between an amount of bronze and a bronze-statue. Causal dependency on the other hand, typically is a relation between fully distinct entities. Because of this difference it would seem that only constitution-based accounts, i.e., Embodied Cognition II and Extended Mind can consider external props as proper parts of the mind or of cognition. The strong parity principle explicitly claims that the mind “extends into the external environment,” i.e., has proper parts which are located in the external environment. This strong parity principle is thus supported by the constitution-based situated cognition theses only. Dependency based accounts on the other hand would have to claim that external props can be ethically on par although they are not a proper part of our cognitive makeup.

Second, constitution is a non-causal, synchronous relation and therefore not suited for any successional relation, which we take causation to be. This difference affects the ethical parity thesis insofar as a constitution-based account can include synchronous extra-neural processes as parts of the cognitive agent only. Causal dependency claims can integrate a larger set of bodily or extrabodily processes into their analysis of cognition or the mind: processes preceding the cognitive or mental processes, their causes. The hypotheses of Embodied Cognition II and Extended Mind will have to limit themselves to bodily or extrabodily processes simultaneous to the intracranial events co-constituting cognitive processes^11^.

Taken these two differences together the best candidates for ethical parity are bodily or extrabodily processes simultaneous to the intracranial events, which according to Embodied Cognition II or Extended Mind co-constitute cognitive processes. Many cases of TBS seem to be part of this candidate group. Especially tDCS, but in many cases TMS as well, is employed simultaneously to the performance of a cognitive task in experimental settings as well as in lay use (Wexler, 2016). As several studies report that task performance is different, sometimes even superior to non-stimulation, it could be inferred that neural processes plus stimulation co-constitute the cognitive process in question.

### The relata: mind and cognition, a processual reading

The analysis of the relations suggested in situated cognition theses has made it possible to limit the *scope* of possible embeddings and proper parts of the mind or of cognition and thus identify candidates for the ethical parity theses. A parallel analysis of the relata will enable us to limit the *types* of possible extensions of cognition. Whether modifications of cognitive processes via either stimulation of neural processes, external cognitive tools, such as pen and paper in multiplication or external carriers of information are possible candidates depends on how we understand the terms “mind” and “cognition.”

While I clearly cannot provide anything like a definition of “mind” or “cognition” in this article, it is crucial to highlight one variation in the use of the terms: “mind” and “cognition” are mostly used either for a set of processes or for some non-processual elements of a thinking being. This distinction can be found in common dictionaries^12^, as well as in philosophical and scientific approaches to the mind.

Note that Walter's definitions above refer to *processes* in the case of cognition as well as its bodily carriers, it does not refer to objects or states in either case. Thus, according to Walter, the primary terms of situated cognition approaches cannot be “belief,” “emotion,” “memory,” or “retina,” “cochlea,” but rather “believe,” “feel,” “remember,” “activate,” “detect” etc. I share Walter's ontological choice, and will try to show why this formulation is superior to alternative formulations, which could for example have been: “Cognitive states are partially dependent upon extracranial bodily states,” or more generally: “Cognition is partially dependent upon extracranial bodily components.” The main reason for deciding for a processual reading is, that it can steer clear of the following problem. In a non-processual reading of the extended mind hypothesis it might make sense to crack the following joke:

Question: Why did the pencil think that 2+2=4? Clark's Answer: Because it was coupled to the mathematician.

(Adams and Aizawa, 2010, p. 67).

Adams and Aizawa have famously put forward the coupling constitution fallacy case against the extended mind hypothesis, which gives rise to above joke. The fallacy consists in identifying two separate objects bringing about an effect together as one system bringing about the effect. The object in question are the mathematician and the pencil, the effect “thinking that 2+2=4.” The coupling-constitution fallacy indeed looms, if we read “mind” or “cognition” as some non-processual elements of a thinking being and insist that some external objects are on par with intracranial elements in constituting the mind^13^. The aim of the joke, Clark as well as Chalmers, often do use formulations which make it seem very much as if they were suggesting exactly this: that external objects are on par with intracranial objects in constituting the further entity mind, and that accordingly, mental states occur in both of these objects alike. The most prominent example is Otto's notebook. In their original contribution introducing the extended mind theory Clark and Chalmers introduce the example of Otto and his notebook. Otto suffers from a memory problem (inadequately dubbed Alzheimer's Disease) preventing him from making use of declarative memories. He uses a notebook instead to gain, store and use information which healthy persons would retrieve from neuraly realized memory (Clark and Chalmers, 1998). In some formulations it sounds as if the individual entries in the notebook are more or less the same as individual memories realized in a healthy person's neural tissue. An ethical parity thesis based on this understanding would claim that Otto's notebook has moral value, because mental states occur in it, i.e., the individual entries are (or are equivalent to) memories.

This interpretation, according to which the notebook is a self-contained realizer of memory, would seem to ignore that the entries in Otto's notebook have a purely derivative cognitive role owed to the notebook being coupled to the cognitive agent. Counter to those formulations of Clark and Chalmers, on a processual reading of “mind” the notebook's entries should not be understood as individual memories, but carriers of a process of remembering. Remembering, however, is not something that occurs in the notebook and neither is the computation 2+2=4 something that occurs in the pencil.

If we take Clark, Chalmers and others to refer to the mind or cognition as sets of processes, the coupling-constitution fallacy does not loom anymore. This is why Shapiro brought forward the charge of a process-constituent fallacy against Adams and Aizawa: “the assumption that those who defend X as a constituent of process Y must believe that process Y occurs in X” (Shapiro, 2011, p. 181).

The procedural reading does, however, have a major influence for the role of the extended mind hypothesis in neuroethics. According to the preferred processual reading, we cannot simply say that Otto's notebook is worthy of protection because memories occur in it. In general it will not do to base ethical inference on the claim that some state occurs in some extracranial or even extrabodily object, even if the ethical inference itself would be sound. This result thus casts doubt on the inference from an extended mind hypothesis to an ethical parity thesis and requires us to think about the ethical relevance of the situated cognition hypothesis anew. Taken together with the result that only processes simultaneous to the cognition to be explained are suited as proper parts of the cognitive agent, the role of situated cognition approaches seems to contract rapidly.

## On the ethical relevance of situated cognition theses

One obvious caveat in evaluating the ethical relevance of situated cognition theses is that we cannot simply move from cognitive to ethical parity (or disparity) theses and vice versa. As just shown it does not immediately follow from the alleged fact that extrabodily processes are constituents of cognitive processes on par with intracranial processes, that they are on par with regard to some moral value, e.g., protection requirements. From the mere fact that Otto's notebook is a carrier of his remembering, it neither follows that memories occur in his notebook^14^ nor that his notebook is as worthy of protection as his neural tissue is. The argument is at best enthymematic as shown initially. On the other hand: neither does it simply follow from an alleged fact that extracranial bodily processes are not co-constituents of cognitive processes, that they are not on par with regard to some moral value, e.g., the claim to bodily integrity. Neither an externalist metaphysical thesis, the extended mind hypothesis, nor an internalist one, the original internal/external distinction, is sufficient for an ethical conclusion. While it is plausible that all constituents of a person's mental life are morally worth of protection, they might well have different protection requirements. And while it is plausible that manipulations of a person's brain give cause for special moral concern, it definitely does not follow, that other manipulations of her body might not be just as problematic, in some cases even more so.

The theoretical distance between situated cognition theses and ethical parity can be demonstrated with one of the frequently used examples. The mere fact that something facilitates or even enables some cognitive process is not a strong indicator for any positive moral value. Take Clark and Chalmers' example of the Tetris support module, no matter whether external or implanted. They ask their reader to imagine an agent sitting in front of a computer screen, trying to predict whether some two-dimensional shapes fit the depicted sockets, i.e., they ask readers to imagine someone playing Tetris (Clark and Chalmers, 1998, p. 8). They provide three methods for the agent: mental rotation of the two-dimensional shapes alone, choice of mental rotation or rotation on the screen, and using a neural implant, which can perform the rotation as fast as the computer. Those three procedures all realize the cognitive process of “rotation.” Should they be functionally equivalent, they are candidates for the ethical parity thesis. And they might well be functionally equivalent: the player can decide to train his mental rotation skills until he beats a less well trained but externally supported or implant-carrying player in one out of ever two games. But why, most ethicists will ask, should a gadget enabling us to better play some computer game have any relevant moral value? There are already special gaming mice and keyboards, sometimes tailored to a specific game. We would not claim that e.g., a special World of Warcraft programmable multibutton mouse has special moral value, because I can solve rotation and navigation of virtual characters faster and more reliably with it, maybe even as fast and reliably as a well-trained and 30 year younger player can with a plain old two button mouse and keyboard. If someone had the idea to implant such a device it would hardly gain in moral value. On the other hand, every gamer's neural tissue, which does enable him amongst other things to navigate virtual characters, has special moral value as a part of an end-in-itself human being's body, capable of happiness and suffering.

Have a similar look at a famous example for embedded cognition: Clark takes up work from McBeath et al. (1995), which explains how outfielders align their movement in order to catch fly balls. He describes how the outfielder does not bother with complex computations of his and the balls trajectory, but rather aligns his visual tracking and his movement until the ball appears to move in a straight line from his perspective. Clark concludes that the solution of the outfielder's tasks is to “maintain a kind of adaptively potent equilibrium that couples the agent and the world together” (Clark, 1999, p. 346). Neither Clark, Levy, Anderson nor anybody else would however—or so I hope—assign the fly ball a moral value even remotely similar to that of the outfielder's arms, legs, or visual and motor cortex.

Thus, situated cognition hypotheses are not sufficient to establish a general ethical parity claim. Neither are they necessary for ethical parity to hold in some cases. Situated cognition approaches can still shed light on the relevance—causal as well as ethical—of extracranial props in human cognition. One will, however, have to take a closer look at the specific external prop or carrier of cognition, in order to establish whether it might be ethically on par with intracranial carriers, have derivative or even no moral value at all.

In the following I will try to provide criteria for such an analysis. First, I'll go into the ways of distinguishing whether a certain prop or tool can reasonably be considered part of an extended mind, a part of the cognitive scaffold or a mere tool (3.1). I will then show that situated cognition approaches do not allow us to differentiate whether the ethical value of some extracranial carrier or modifier of cognition is original or derivative (3.2). In support of that I'll demonstrate how even the cognitive role of most technological tools including tBS are —counter to the demands of the ethical parity thesis—not functionally equivalent, but rather functionally complementary (3.3). As complementary carriers of cognitive processes, even their cognitive role typically is derivative of that of our biological make-up. In conclusion I will provide a criterion which is suited to distinguish between primary and derivative moral value of carriers of cognition as well as providing support the criteria established in 3.1 to distinguish between tools, scaffolds and proper parts of cognition (3.4).

Based on these criteria it turns out that several cognitive and neurotechnologies which have been considered part of an extended cognition are better described as mere tools or scaffolds, though it is still plausible to assign them a relevant moral value.

### Mind-extension, scaffold, or mere tool?

As detailed above, situated cognition theories, with the possible exception of Clark and Chalmers' version of the extended mind hypothesis, were not originally targeting cognitive technologies. Brain stimulation technologies did not feature even in Clark and Chalmers' version. It is not at all clear, whether cognitive technologies are best described as a proper part of the extended mind, a part of the mind's scaffold or as a mere tool.

From early on authors of situated cognition theses have been acutely aware that one needs to distinguish mere tools, external scaffolds of cognition and external carriers of cognitive processes. The difference has been marked by Clark and Chalmers, by what later came to be known as “glue and trust,” that external carriers of cognition have a set of distinctive characteristics distinguishing them from mere tools. Their requirements can be summarized as the external carriers of cognition being a constant in the person‘s life, being directly available without difficulty, their information becoming automatically endorsed by the user and having been consciously endorsed prior to that (Clark and Chalmers, 1998). Others have developed further criteria or dimensions of integration between cognitive agent and external carriers. Sutton et al. for example present a dimensional analysis integrating reliability, durability and the criteria of glue and trust as presented by Clark and Chalmers (Sutton et al., 2010). Menary adds dimensions of manipulation and transformation (Menary, 2010).

In a number of recent articles Heersmink (2015) has taken up the debate about “glue and trust” and provided an extensive set of criteria which are meant to distinguish parts of the extended mind from scaffolds and maybe even mere tools. He suggests evaluating a cognitive artifact on the following dimensions: (1) information flow, (2) reliability, (3) durability, (4) trust, (5) procedural transparency, (6) informational transparency, (7) individualization, and (8) transformation. Some of these dimensions seem to be custom made for fairly specific cases of modern digital lifestyle appliances i.e., wearables, smartphones and tablets. Especially information flow, trust and informational transparency, criteria designed for tools and props containing representational information, are of limited use for scaffolds and props which are not even intended to carry information, such as brain stimulation technologies or pharmaceuticals (cf. Heersmink, 2015, p. 583 f. and 589 f.).

When evaluating new neurotechnologies, especially brain stimulation technologies, these criteria should give us pause. Remember that our investigation started, because according to Levy and others “[a]ccepting the extended mind thesis […] requires us to reject […] that interventions into *the brain* are uniquely and distinctively interventions into the mental states that constitute our identities” (Levy, 2007b, p. 7). Rather they are *qua* extended mind hypothesis on par, ethically and functionally, with “using traditional psychological methods, which range from talk therapy to the complex experimental manipulations of social and cognitive psychologists” (Levy, 2007b, p. 7). Based on the above criteria, however, it is highly dubious whether current technologies for transcranially intervening in the brain are cases of situated cognition at all:

As mentioned, the criteria information flow, trust^15^ and informational transparency are not applicable to brain stimulation devices. Although those can affect the information processing and maybe even information retrieval of a subject, this is—at present—not an intended nor a reproducible effect of these technologies. This is one obvious difference between brain stimulation technologies—as they exist today and in foreseeable future—and several other modifications of the mind: brain stimulation technologies are not content evaluable^16^.

On the remaining dimensions as discussed by Heersmink, tBS technologies typically score fairly low. Note that the following evaluation refers to transcranial stimulation only; other stimulation neurotechnologies, especially therapeutic Deep Brain Stimulations and Vagus Nerve Stimulation, are much better candidates for mind extensions. Should these fail the test for inclusion into the extended mind, then because their effects are less cognitive than motoric.

While several lay users claim otherwise, the effects of current transcranial stimulations are fairly minimal and strongly dependent on precise circumstances, ideally laboratory circumstances (Horvath et al., 2014). While current research into—not with—tDCS and TMS aims at creating a reliable manipulation of cognitive and other mental states (cf. Heinrichs, 2012) current *reliability* is comparatively low.

Brain stimulation technologies are not a *constant in a person's* life, not even her or his cognitive life. Rather, current stimulation technologies are used in rare occasions only. Furthermore, a constant use is explicitly advised against by the relevant expert consensus statements (Rossi et al., 2009). While it is possible that some lay users exaggerate their use of tDCS, we have yet to see people constantly using any such tool. Thus, uses of brain stimulation technologies—at least transcranial ones—typically are of limited duration and repetition.

Current stimulation technologies are extremely cumbersome to use, making them rank really low on *procedural transparency*. Effective TMS devices are bulky, require quite some energy and most of all, require a fixed targeting system. Ideally, TMS is applied stereotactically after the target area has been identified by a MRI scan and correction for deviation between standard brain models and the individual brain. tDCS-devices are admittedly much smaller and require less energy, but to realize a measurable and specific effect, one needs at least to securely place electrodes on the scalp and limit the scalps conductance by keeping it clean and free from sweat and hair. Identifying a correct target area again is not as simple as it sometimes is depicted. As individual brains differ, the likelihood of realizing the desired effect decreases dramatically if no MRI scan and appropriate computation of loci is present. As movement is detrimental to stable electrode positioning, the whole process needs to take place in a nearly immobile state. The procedural transparency of sticking electrodes on your scalp and keeping immobile is far from what one would require of a part of the extended mind, even of the normal embedding of a cognitive agent.

The criteria of individualization and transformation are not easily applied. While in the times of tDCS-tinkering the individualization of self-made devices is extremely high, this is more an effect of the manufacturing infrastructure and less one of adoption to one's individual needs. While the stimulation program especially of TMS can be adapted to the individual's physiology and reaction, this is not a case of *individualizing* the device, but rather of using task-adequate settings. We would not consider a coffee bean grinder an individualized device either just because it can be set to fine or coarse grind.

The *transformational* power of stimulation devices is limited in duration. They certainly change the short-time behavior of stimulated areas, but long term effects are hard to come by, even with many sessions of rTMS. Furthermore, stimulation technologies tend to modify the short-time performance of the cognitive system more than the structure thereof. Typical effects users look for and claim to have realized are rather of the “more x” type, e.g., more creativity, more concentration, more relaxation. Changes in the mode and structure of cognition tend not to be sought nor found. Thus, the transformative power of stimulation technologies now and in the foreseeable future is rather limited.

These eight criteria can and should be supplemented by a ninth, replaceability. I want to suggest that an artifact is more integrated into a joint cognitive system, if it cannot be replaced or can only be replaced by similar artifacts without abandoning the cognitive process in question. As this criterion plays a dominant role in the ethical analysis as well as a dimension of integration, I'll discuss it in detail below.

With a low score on five applicable criteria and three criteria simply not relevant for this type of technology, tBS is not a good candidate for being a proper part of an extended mind. It even is alien to the typical embedding of our cognition. As will become apparent in the following, the same result holds on the additional dimension of replaceability. This second negative result complements the first: as mentioned above, situated cognition theses are neither necessary nor sufficient to establish ethical parity between intracranial carriers of cognition, extracranial ones, their scaffold or cognitive tools. Now it turns out, that a major part of new neurotechnologies, namely transcranial stimulation technologies, are not part of our extended mind or its scaffold but rather external tools for modifying mental states. The double negative does not simply make a positive in this case either. As Levy and Anderson claim, cognitive technologies and tools can be ethically on par with neural carriers of cognition, but this neither follows from situated cognition theses alone, nor is it the case in general. Rather one will have to analyze the specific composition of neural and non-neural carriers of a specific cognitive process in order to evaluate their moral standing, e.g., their worthiness of protection or whether there might even be a claim for their social provision.

### Situated cognition and derivative moral value?

If the situated cognition thesis were ethically relevant it should make a difference for the evaluation of ethical parity under which specific version of the situated cognition thesis a prop can be subsumed. It should not merely affect the scope of the allegedly morally relevant cognitive system: bodily or extrabodily processes, one would expect it to affect the type of moral value ascribed. Constitution-based accounts (EC-II or EXC) make the external processes a real part of the cognitive system, in the specific case of the cognizing human agent or human-prop system. As such it would at least be plausible to ascribe it the primary moral value which human agents have, i.e., participation in her or his moral status. The causal dependency versions (EC-I and EMC) in contrast, should leave the external processes as non-constitutive, i.e., they should accept that the cognizing agent and its cognitive processes depend on something which is not a proper part thereof. The moral value which one would expect to be ascribed to the external prop in this scenario is derivative to the moral status of the cognizing human agent.

However, this difference between constitution and dependency-based account does not obtain: *all* moral value of tools, scaffolds and even mind extensions is derivative. The moral value even of mind-extensions—if there are any—depends on the extension being a part of a joint cognitive system with another individual, which enjoys unconditional moral value independently of the extension. If Otto's notebook were a mind extension, it would enjoy moral value only because of its contribution to the cognitive system notebook-Otto, in which Otto has moral standing, and Otto has moral standing independently of his notebook. Otto on the other hand does not enjoy his moral standing because he contributes to the cognitive system Otto-notebook. Moral value in this as in all other mind extension or scaffold cases is asymmetric.

This is why interpretations of *embodied* cognition theses cannot add to the moral value of the extracranial carrier or scaffold of cognition. Embodied cognition theses refer to proper parts of the cognitive agent's body. A cognitive agent's body already enjoys the highest moral value possible, it need not be inferred from an embodied cognition hypothesis. The only additional ethical claims can, therefore, refer to extrabodily processes, which can have a role as carriers or conditions of cognitive processes.

Some of our external tools and scaffolds enable or facilitate cognitive processes in the same way as external carriers of cognitive processes, and while a token of a specific process is ongoing, manipulation of the tool will typically disrupt the cognitive process. Accordingly, their derivative moral value can be extremely high, such as being the content of an agent's absolute claim-right, but it will always be derivative of the moral value of the cognitive agent himself.

### Complementarity and equivalence

An ethically relevant difference between the diverse external contributors to cognition can be found in how they are entangled with the internal contributors in a specific process. Two different types of entanglement found attention in situated cognition approaches and in the ethical parity theses: complementarity and functional equivalence.

Situated cognition as a paradigm of the cognitive sciences mostly aims at explaining how cognitive processes are realized by *complementary* neural and extracranial processes. Only in a few cases, such as Clark and Chalmers' example of Otto's notebook, did a situated cognition approach focus on external carriers of cognition which perform the same or an *functionally equivalent* task as the intracranial carriers. Even one of the authors of this example, Clark, assigns priority to non-functionally equivalent but *complementary* external resources (cf. Sutton et al., 2010, p. 524 f.). Consequently, the main examples of situated cognition approaches have been structures in the environment allowing a reduction of the agent's cognitive load, or mechanisms in the body enabling or facilitating processes of cognition.

The ethical parity theses on the other hand aimed at *functionally equivalent* neural and extracranial carriers of cognitive processes^17^. Prostheses for therapeutic and enhancement goals that aim to replace or reproduce cognitive processes usually realized in neural matter have been their main topic. The authors behind the ethical parity theses refer to functional equivalence in their discussion, but turn to functionally complementary tools in their examples. As already mentioned, Levy primarily refers to psychopharmaceuticals, brain stimulation devices, computational devices, such as PDAs, all of which perform complementary or modifying functions, not equivalent functions. In the same vein, the examples that Anderson employs in his introduction of the ethical parity thesis are not cases of replacement and functional equivalents but rather cases of complementary functions. His protagonist uses either a PDA or an implanted device enabling her to hear and analyze frequencies, which she could not hear with her normal biological endowments. Therefore, it is likely that Anderson would agree to extend his ethical parity thesis to functionally complementary carriers of cognition.

It is, however, at least plausible to think that it does make an ethical difference whether a external scaffold or carrier of cognition complements the intracranial carrier or replaces it within the cognitive agent. Early examples used by embedded cognition theorists have been structures in our common environment, such as trajectories and velocities of flying objects (see Clark's example of the outfielder above). It is hardly plausible to formulate ethical parity theses equating the neural carriers of visual perception in motion and the environment the perceiving agent moves around in, especially the baseball which Clark's outfielder is going to catch (Clark, 1999).

Even after accepting that the moral value of mind extensions and scaffolds is merely derivative, there still seems to be a huge gap between the moral value of the outfielders visual and motor cortex and the fly ball. The fly ball's moral value—if it has any at all—is not similar to, if derivative from, that of the outfielder's brain. Interfering with the fly ball's trajectory would merely violate the rules of the game, interfering with the visual and motor cortex of the outfielder would likely be a case of criminal assault.

Thus the ethical parity thesis seems to require either a limited scope—limited to those external carriers of cognition which play a functionally equivalent role. Or it is open for an internal differentiation assigning equivalent ethical value to functionally equivalent external carriers of cognition and some type of corresponding ethical value to functionally corresponding external props. An external prop which fulfills a complementary role is in most cases irreplaceable in the cognitive process token. You cannot simply switch the fly ball in mid-run. Throwing an additional ball into the game would not merely break the rules, it would confuse the outfielder's cognitive process. It often is, however, replaceable in the process type. Next inning, the outfielder's cognitive process type will be complemented by another ball. Functionally equivalent external props are however often, if not always, intended to work stably over time and process tokens. Thus, the difference made by complementarity or functional equivalence might even be reducible to irreplaceability, as discussed below.

This is one major point in analyzing the moral value of brain stimulation technologies, such as TMS and tDCS. Most of them, especially in non-therapeutic settings, perform a complementary function, thus are not functionally equivalent to internal carriers of cognition, if they can be seen as carriers of cognition at all^18^.

### Replaceability

The inference from an external prop's role in a cognitive process to its—always derivative—moral value depends among other things on the specific role—equivalent or complementary—played in the process in question. The main characteristic of a prop's role is its replaceability. Replaceability can refer to two distinct but related possibilities: the possibility of immediately replacing a certain contributor in the ongoing token process or the possibility of replacing the contributor in future process tokens of a specific type long-term. It is for example possible to *immediately* replace my edition of Aristotle's works by any other paginated edition during the process of my counting how often he used the word “representation.” If you swap the books open at the same place of the text fast enough I will hardly hesitate. It is equally possible to replace my use of pencil and paper for multiplication in the *long-term* if one provides a number of ten-sided dice and some time for practice.

With “replaceability” in both cases I refer to real replaceability here and now. While one can always make up some science fiction scenario in which some future replacement technology is possible, that is not what I refer to. Binding replaceability to a state of development does mean that moral value can change over time and with technological development. In some future high-tech scenario the blind man's cane might be a relic, something that is extremely limiting and therefore offering it to a blind man instead of his high-definition, low light and infrared supporting retina replacements a moral affront. Here and now it is not an affront but a sensory extension, which it would be morally blameworthy to interfere with, much less damage or take away.

Replaceability in the long-term is not merely at the heart of the difference between tool, scaffold and mind extension, it is at the heart of ethical value as well. Generally, an irreplaceable contributor to one and the same cognitive process is *ceteris paribus* morally more important than a replaceable one. Because the loss of an irreplaceable contributor to a cognitive process often is the loss of an ability of the cognitive agent, it makes sense to count the external contributor as a part of his cognitive makeup, his mind.

Thus, if there are external carriers of cognitive processes which cannot be replaced by either internal carriers or other external ones, they are the best candidates for a moral value that is equivalent to, if derivative of the one currently enjoyed by internal carriers (more on the “currently” below). This constellation is especially plausible for embodied cognition, as some bodily carriers, especially in perception, seem as of yet not to be replaceable, e.g., the ability to modify the visual perspective by bodily movement. It is harder to think of an example of embedded cognition with an irreplaceable external contributor.

An external technical carrier of cognition would similarly have to be irreplaceable by internal carriers or other external carriers to gain a similar standing. Imagine a cochlea-implant like hearing aid implanted early in an individual's life. Let us say it cannot be replaced later in life for whatever techno-biological reasons. Such an implant would be just as worthy of protection as a natural cochlea. A person who had such an implant in his left ear and a natural grown cochlea in his right would be equally hurt if either of them were tampered with.

An ability requires an external scaffold, if its performance requires an external contributor, which however can be replaced by alternatives of the same or a similar type. The specific scaffold used in the process token can be replaced without the ability being lost. These external contributors of cognition will have more than a modulating role. They are replaceable by other scaffolds, i.e., the process in question could not take place without some external contributor to cognition, but it does not have to be this specific one, maybe not even one closely similar. The famous notebook of the deeply forgetful Otto seems to belong into this category, as do pen and paper in long multiplications. Such a scaffold can either be replaceable by a similar, that is functionally equivalent or by a non-similar prop. In the latter case the same cognitive process can be realized by different combinations of neural matters and external hardware. Otto needs not have used a notebook, he could—as in the movie Memento—have taken recourse to tattoos, or, closer to current therapeutic practice in dementia cases, to imagery, photos, SenseCam shots.

The remaining external contributors, which can be replaced by purely internal carriers of the process, are mere tools. These external carriers of cognition have a merely modulating role in a cognitive process, i.e., the process in question could and would take place without them. The specific way it currently takes place, however, is determined by the props. The use of paper and pencil in adding sums seem to be such a case. Most people would be able to add sums without external carriers of cognition, but they actually do it using a scrap of paper and a pen. If a certain external carrier of cognition can be replaced by an internal mechanism, then it is a mere modulating tool.

At the current state of development, tBS technologies belong in this category of mere tools, if in any of the above. TBS as an external tool always has a derivative moral value if any. Its role is complementary, meaning that it is not plausible to assign it an equivalent moral value to that of the stimulated neural carriers of cognition. There is of yet no cognitive process that cannot be realized without the help of tBS, rather tBS facilitates common cognitive processes, but could—long-term and sometimes even immediately—be replaced by the inner workings of neural tissue^19^.

If replaceability makes the difference between tool, scaffold and mind extensions as well as the difference in moral value, does that not mean that a situated cognition approach, the extended mind hypothesis, is back in the ethical boat? Are not mind extensions morally of the highest importance according to this argument? This conclusion is suggestive but wrong nevertheless. It is wrong because firstly the moral value assigned to all of the external contributors to cognition is still derivative, thus ethical parity is out of the question from the beginning. Second, that mind extensions have a stronger moral value than scaffolds or mere tools does not mean they have the same moral value as an internal carrier of cognition. While that might come to be the case—see below—it currently just isn't. Third, sharing a criterion does not make a metaphysical and an ethical status any closer related. That replaceability is one criterion for the integration in a cognitive system as well as for the moral value of an external prop does not make the integration into a cognitive system a moral value or a reason for moral value.

The criterion of replaceability is not an *ad hoc* suggestion, suited to external props only, no *lex technica*. It can be applied to neural carriers of cognitive processes and doing so reveals some interesting results. First of all, if ethically less relevantly, there are some internal carriers of specific cognitive processes which can be replaced by alternative internal carriers. That typically happens if functions originally realized by one neural area are taken over by others after some kind of damage, such as a stroke. As proper parts of cognitive agents performing the same function at different points in time, these are more or less obviously morally on par. What is ethically more interesting, however, is the change in moral value, which does arise through workable external replacements. Following Anderson we ought to value different embodiments of a human being just as we do the average embodiment, because the main target of respect is not the specific body but the human being and her cognitive processes. Thus, with the development of functional external replacements at least the uniqueness of the moral value of internal carriers of cognition dissolves.

That does indeed open up a route to ethical parity. The route to this parity is highlighted by the dimensions of integration as discussed by Heersmink and the additional dimension of replaceability: if an external tool does become a closely integrated, irreplaceable carrier of some cognitive processes, it will be nearly ethically on par with internal carriers of the same process. The alteration of either of them would be an assault of the same gravity on the cognitive life of the agent. It would, however not be ethically on par in other regards: the internal carriers would still enjoy the additional protection of a proper part of a human being, which does not depend on or comes to the same as the protection of a carrier of cognitive processes. We do not merely protect the cognitive or mental integrity of humans, we do protect, even primarily, their bodily integrity.

At the moment most technologies, especially brain stimulation technologies are far from this state of development. For brain stimulation technologies it does mean that their current development path toward more stability, reliability and specificity, which is in the medical and scientific interest, will not be sufficient to include them in their user's extended mind. Rather the unwieldy tools, which they currently are, will have to be turned into operationally transparent, constantly useable devices. What is more, they need to enable cognitive processes beyond what is possible without them. This is at the same time what “enhancement” ought to be about and what makes an external contributor of cognition a morally relevant object: enabling new or radically improved cognitive functions.

## Moral value without ethical parity without situated cognition

Note that Levy's and Anderson's claim of ethical parity is merely a negative result itself: it is *not* the case that the internal/external distinction as such plays a significant ethical role in the evaluation of influences on the mind. The parity thesis does not provide any positive reasons for the evaluation of specific tools, scaffolds or mind extensions. As mentioned initially, I share Levy's and Anderson's conviction that external and internal modifications of cognitive and mental states can be ethically on par. As shown above, this parity is not a consequence of the situatedness or extension of cognition. Neither is the metaphysical thesis that external tools are *not* a part of the mind suited to guide neuroethics, nor can the opposite metaphysical interpretation of situated cognition approaches guide neuroethics. This guidance can only be performed by specific role of individual tools, scaffolds or extensions in a specific cognitive process. In this I agree with Levy: “the very same reasons we have to fear neuroscientific mind reading and mind control apply, with at least equal force, to existing techniques, and perhaps even more to new discoveries coming not from neuroscience but from cognitive and social psychology” (Levy, 2007a, p. 155).

The same moral reasons can apply to all the components of a cognitive process, depending on their role in the cognitive process, but independently of their location, i.e., in the brain, in the body or in the environment. To that extent the weak ethical parity thesis, unlike its strong counterpart, holds. However, at present?, some moral reasons are adequately applied to internal carriers of cognition and not to external ones. That will only change if external carriers of cognition become even more deeply integrated into our cognitive makeup and become irreplaceable conditions of specific cognitive functions. Even then, however, the main ethical task will remain open: providing the moral reasons, reasons appealing to instrumentalisation, to adverse consequences, to authenticity, to personal identity etc., for or against a certain manipulation of cognitive processes, by whatever means.

## Author contributions

The author confirms being the sole contributor of this work and approved it for publication.

### Conflict of interest statement

The author declares that the research was conducted in the absence of any commercial or financial relationships that could be construed as a potential conflict of interest.

## Footnotes

^1^For an overview of the coverage transcranial stimulation received and the overly strong claims made in that coverage see (Dubljevic et al., 2014), for the similarity in coverage concerning transcranial stimulation and psychosurgery see (Mashour et al., 2005)

^2^For a detailed discussion of Levy's EPP see (DeMarco and Ford, 2014). DeMarco and Ford suggest an even weaker form of EPP, which I will not include in the following argument for the sake of brevity.

^3^One might read this as a global attack against neuroethics: If neurotechnologies are ethically on par with established technologies and the additional risks of neurotechnologies are ethically no different from those of other medical operations, then one might think that the ethical principles which stood the test of time in evaluating established technologies in combination with the bioethical principles are sufficient to evaluate new neurotechnologies as well. Levy obviously does not read his argument this way.

^4^Prior manuscripts of Anderson's paper date to 2007.

^5^Anderson takes causal dependence to be the criterion, which makes external carriers of cognition part of a system, which solves cognitive tasks: “Since I cannot do the arithmetic without the paper and pencil (or calculator), it seems clear that they are essential parts of whatever system it is that is doing the long division.” (p. 264) This description is compatible with embedded cognition approaches as well as with the extended mind hypothesis.

^6^Walter claims that BCI might at best be discussed under the paradigm of *enacted cognition* I take *enacted cognition* or *enactivism* to be a close relative of situated cognition hypothesis. It claims that cognition always is enacted by an embodied cognitive system in and with its environment. Thus it combines the embodiment and embeddedness thesis, but goes beyond those in requiring that the cognitive system in some way makes sense of the relation between itself and the environment in the performance of cognitive processes (cf. e.g., Hutto, 2008, 2013). The reason why enactivism is not discussed in this article is simple: it has a strong anti-cognitivist flavor. Enactivism at least in its more radical strands tries to reduce cognitive processes to simple, where possible motoric and perceptual, routines.

^7^This critical note can be found in Levy's own work: “I also suggested that nothing of great moment depends, for our purposes, on whether it or its less radical rival, the embedded mind hypothesis, is true.” (Levy, 2007a, p. 308) It is mirrored in the contributions of e.g., Sterelny (2010), Sutton (Sutton et al., 2010) and others.

^8^Recent suggestions, however, cast doubt on the assumption that computationalism and the embodiment thesis are mutually exclusive. In fact, from a computational perspective, the spread of the computational hardware is not limited in principle (Wilson, 1994), and from an embodiment perspective nothing speaks against identifying the processes of the wider human body as computational processes. A distinction of situated cognition theses similar to Walter's is made by Shapiro, who takes the viable embodiment programs to either be a case of replacement or of constitution. It should be noticed that Shapiro's discussion of embodied cognition includes theoretical approaches typically subsumed under embedded or extended cognition theories, namely the dependency or constitution of cognitive processes by extrabodily components (Shapiro, 2011).

^9^According to embedded cognition theorists, the cognitive load allegedly caused by representations can be reduced by relying on the world as its own model. Embedded cognition theories owe a lot to the robotics program of Brooks (1991), who designed several of his early robotic systems as artefactual replies to the early artificial intelligence program, even in their names. Brooks' robotics program, apart from setting trends in robotics, highlights the compatibility of different situated cognition approaches.

^10^Constitution has been the focus of a long debate raging over different contexts, from personal identity to mechanistic explanation (Baker, 2000; Craver, 2009). I'll follow Carl Gillett in his characterization of constitution as “(c)ompositional relations” i.e “non-causal determination relations that are synchronous, involve entities that are not wholly distinct and do not involve the transfer of energy and/or mediation of force. Scientific relations of composition are also (b) transitive, asymmetric and irreflexive and (c) many-one relations with teams of many components and one composed entity.“ (Gillett, 2013, p. 317) Gillett provides a much more detailed discussion of constitution, focused on explanation in the sciences.

^11^One reason why authors in situated cognition theory exclude technological artefacts is to stop a *reductio* argument against the situated cognition theses, called *cognitive bloat*. The *cognitive bloat* argument claims that once one accepts external carriers of cognition as part of the mind, there is no bar to extending the mind even further, and soon one will have to accept every accessible book, website, possible social interaction etc. as part of the individual's mind. As this is absurd, one had better not even take the first step and reject the extension thesis altogether. With a broad scope of external parts of the mind it would be at least extremely difficult to give a unified account of all the different components of a mind so understood. The term would simply refer to seemingly unrelated stuff. The same broad understanding of situated cognition raises a similar problem for approaches using a dependency account. Widening the perspective by including too much of the causal history of a cognitive process will make it increasingly difficult to find unified explanations. For a paradigm intended to provide greater unity to explanations of cognition, this would be unfortunate. This has been brought forward as a critique to situated cognition theories in general: extending the explanandum cognition‘beyond what goes on in the brain will make it harder or even impossible to find an unified, scientific theory (Rupert, 2004; Adams and Aizawa, 2008). The argument is peculiar: not only is it one of the infamous arguments from the future progress of science, rather it tailors the phenomena to some future theory instead of tailoring the theory to the phenomena. Levy provides a detailed discussion of this issue in his (Levy, 2007a, p. 48 f.), pointing to existing scientific theories of very divers phenomena. But see the reply in (Adams and Aizawa, 2010) as well.

^12^The Merriam Webster for example gives three definitions of the noun, mind' as used in this context: (a) the element or complex of elements in an individual that feels, perceives, thinks, wills, and especially reasons, (b) the conscious mental events and capabilities in an organism, (c) the organized conscious and unconscious adaptive mental activity of an organism. Definition *a* refers to a non-processual, b and c to an processual conception of, mind'.

^13^Even then, the joke has a taste of a mereological fallacy (cf. Bennett and Hacker, 2003): it is not the case that a part of a system can be assigned the same properties as the complete system, e.g., it is not the case that the brain thinks, just because persons with a brain think.

^14^A good example for this–to my mind dubious–inference can be found in (Sutton, 2010, p. 195): “When extended from cognitive processes to cognitive states, it also then animates the stronger claim that the *standing* information in the notebook counts as cognitive even when it's not in use, because it functions in relevant respects just as do the standing, non-occurrent beliefs and memories in Inga's brain.”

^15^“Trust” is introduced as trust in the device's *information*. One could reinterpret it as trust in the device's effect at best. That, however, does not seem to catch the particular *cognitive* role of external devices.

^16^Both, TMS and tDCS can enhance language performance including semantic processing (Andoh et al., 2006), word associations (Bridgers and Delaney, 1989) or picture naming (Cappa et al., 2002; Sparing et al., 2008). As has been well known for decades, electric cortex.stimulation can elicit the retrieval of contentful memories, not however generate new contentful states nor selectively activate specific contents, unless by replicating prior chance findings (Penfield, 1958).

^17^(For the difference between parity and complementarity-based Extended Mind theories cf. Sutton, 2010, p. 193 f.)

^18^rTMS is approved for treating Major Depressive Disorder in adults who have not responded to prior antidepressant medications (Perera et al., 2016). In this setting it seems to restore a function, which the healthy brain typically performs. While there is obviously a cognitive effect in the treatment of depression, this is not a counterexample to the complementary character of transcranial stimulation technologies. rTMS does not perform the function, which the healthy brain would have performed, thus does not replicate or replace the latters function. Rather it modifies the brain of the person suffering from depression in such a way that it can perform the same function as a healthy person's brain.

^19^In the case of treatment of major depressive disorder via rTMS, this even is the goal of the procedure. rTMS is not employed in order to alleviate depressive symptoms during stimulation only. Rather the intention is to modify the activity of the brain beyond the duration of active stimulation.
